# The New York Institute of Stomatology

**Published:** 1905-12

**Authors:** 

**Affiliations:** The New York Institute of Stomatology


### Reports of Society Meetings.

---

### THE NEW YORK INSTITUTE OF STOMATOLOGY.

At a regular meeting of the Institute, held Tuesday evening, October 3, 1905, at the "Chelsea," 222 West Twenty-third Street, Manhattan, a paper was read by Dr. Charles McManus, of Hartford, on the history of Dentistry from the earliest times to the founding of the profession by Hayden and Harris, with special reference to the immediate publication of such a work in English.

(For Dr. McManus' paper see page 826.)

A meeting of the Institute was held at the Chelsea, No. 222 West 23rd Street, New York, on Friday evening, November 3rd, 1905, the president, Dr. C. O. Kimball, in the chair.

The minutes of the last meeting were read and approved.

Dr. Fossume presented for the inspection of the members some samples of removable bridgework.

Models were also presented of the jaw of a girl thirteen years of age who erupted the upper bicuspids in a completely reversed position, *i. e.*, having the lingual cusps outside.

The paper of the evening was presented, with accompanying lantern illustrations, by Dr. L. W. Baker, of Boston.

(For Dr. Baker's paper see page 784.)

*Dr. S. E. Davenport.*—It has been the privilege of the institute during the last three years to present some of the ablest orthodontists in this country. To-night we have had another valuable contribution to this subject, the second one from the Baker family. We feel under great obligations to young Dr. Baker for what he has done for us.

*Dr. C. D. Cook.*—I would like to ask Dr. Baker what influence parentage has in these cases of irregularity. We hear a great deal about the child inheriting the large teeth of one side of the family and the little mouth of the other with a resulting crowding of the teeth.

*Dr. Baker.*—I suppose heredity may have something to do with these malformations, but on the other hand, did you ever see one of these same children with a malformed hand or any other malformation from a cause similar to that mentioned by Dr. Cook? It hardly seems to me that nature would permit of such a discrepancy.

*Dr. H. L. Wheeler.*—I think there is absolutely no comparison between the development of the hands and that of the teeth. I believe that one of the reasons why the teeth are irregular and imperfectly developed is lack of use. Change in our methods of preparing and taking foods has begun to interfere with the development of the teeth. A degenerative influence has taken place because of a diminution of the work required by the jaws. This change has not taken place with regard to the work required of the hands.

*Dr. F. Milton Smith.*—Will Dr. Wheeler kindly explain according to this theory why it is that I have a perfectly regular arch while that of my brother, born a year and nine months before, has a very contracted arch and very irregular teeth.

*Dr. Wheeler.*—In a specific case I should have to know the history of the family and the environment to give any satisfactory explanation. Nature always endeavors to adapt herself to her environment but if the environment changes rapidly there may be an interval when nature will fail to succeed in her perfect adaptation.

*Dr. Wilbur F. Daly.*—It seems to me in all these discussions the deciduous set of teeth have been entirely overlooked. I believe that if the temporary teeth are prematurely lost we are bound to have a contracted arch. If they are retained normally we are bound always to have a normal set of permanent teeth.

*Dr. Baker.*—How about cases of mouth breathing where there is almost always a narrowing of the arch.

*Dr. Daly.*—The mouth breathing comes from enlarged tonsils and adenoids. These will not appear if the temporary teeth are normally retained.

*Dr. E. A. Bogue.*—I was sorry to see the discussion wandering off the subject as it did. I think perhaps what Dr. Daly meant to imply was that the deciduous teeth, just as accurately as the permanent teeth, will tell the story of impending irregularity. If he has not got as far as that, allow me to say that such is the case.

Dr. Baker asks (after showing a number of interesting specimens where there seemed to be an improvement in intelligent appearance), "Has a psychological change taken place?" As he has asked the question, it seems to me that we may try to answer it. I think such a change has taken place. I not only think so but I could point to a dozen authors who have come to the verge of this discovery,—Dr. G. V. I. Brown, Milwaukee; Dr. Swayne, New Haven; Dr. Behrens, New York, and many others.

Dr. Pfaff, of Dresden, Germany, read a paper at the International Dental Congress at St. Louis, containing the statement that partial occlusions of the nasal meatus were corrected by enlargement of the superior dental arch. In the fifteen hundred examinations or thereabouts that I have made I have found a great many cases carrying out the view that if the superior arch is regular the nasal meatuses will be regular also. In regard to lower arches the rule is that the lower teeth erupt first forming a mould into which the upper teeth erupt. There are exceptions to this rule. By just so much as the dental arch is regular, the arch of the palate regular, the palate bones sufficiently large, the sphenoid and the ethmoid bones normal, by just so much will all the cavities of the nose and pharynx be large and resonant. Now, if in a case of contracted arch this contraction is corrected, the broadening and enlarging of all these bones with a consequent enlargement of the various adjacent cavities, makes it reasonable to suppose that there is more or less of an enlargement of the cavity in which rests a portion of the brain. While I am no believer in the so-called science of "phrenology" I have no doubt but that the development in early youth of these upper maxillary bones adjacent to the brain does increase the mental capacity, and I think perhaps this explains the phenomena so nicely shown in Dr. Baker's demonstration, and presupposes a psychological change.

It is of course desirable to treat these cases of regulating as promptly as possible, so as to make use of the temporary teeth to attach the regulating fixtures. It was not ten days ago that I heard a scathing criticism of a number of cases where decay of the permanent teeth had resulted from the appliances. So I want to raise my voice against the use of regulating rings without cement. I recognize also the desirability of getting these appliances off as quickly as possible with safety to our work.

Even after we have done all that is possible in the correction of an irregularity, time must be allowed for nature to grow up to the new condition. When we say that in a few weeks we have finished a piece of regulating, we are going farther than the results will permit. Accurate retention for a certain time is absolutely necessary. I remember in one instance saying to Dr. Baker, Sr., after seeing an appliance that he had adjusted for retaining a set of teeth, that he was retaining them in an abnormal position. He acknowledged this after the retainer had been removed and nature had had time to readjust the teeth into their proper positions, the cusps occupying the sulci of the antagonizing teeth.

I want to make my acknowledgements to Dr. Angle for his classification, and also to Dr. I. B. Davenport of Paris, who in the Cosmos of July, 1887, published the first scientific article on the occlusion of the human teeth. It was this work I am told that gave Dr. Angle his first incentive in scientific orthodontia. Dr. Baker alludes to three forces, among them the expansion arch which is more than one hundred years old. He alludes to the screw. This was invented by Mr. Archimedes several centuries ago. Then he alludes to ligatures. I do not know who was the originator of these. A great deal has been accomplished by a good many men, adapting these three forms of force and that of elasticity to the regulation of teeth. I do not think that anyone has done entirely well nor anyone entirely ill. Perhaps later we shall produce an apparatus that will be far more effective than any we have yet.

*Dr. Baker.*—Do you suppose such an appliance can ever be discovered?

*Dr. Bogue.*—It is not impossible. It is not the Angle, the Coffin, the Ainsworth or the Jackson appliances, but each one of them contributing to the understanding of the *principles* underlying orthodontia, and a valuable something to the mechanical part of it that brings about advancement in the science. No apparatus in the hands of an uninformed dentist will or can be efficient.

In every one of his cases Dr. Baker alluded to the widening of the arch. There was not an exception. What does this mean? We see advertised very extensively apparatus for drawing the teeth back. How often is that needed I wonder. Every one of the cases shown us to-night was defective because there was a lack in the upper maxillary region. It shows in the nose, lip and molar region, as well as in a weak chin. I think if you will observe closely, you will find that one third of the people you meet on the streets are defective in some respect in this region and in a way that could have been corrected with proper attention early in life.

Dr. Baker mentioned that if teeth were corrected to their normal positions they would be retained by the force of occlusion after a little time had elapsed. I think the president will bear me out in the statement that was made years ago that if the lower teeth were made properly to occlude with the upper and were kept in their places, no retaining plate would be necessary for the upper teeth.

Dr. Baker also said that extraction was indicated once in a while. So is the amputation of a leg and just about as often. I have taken out one tooth for one young lady, an upper lateral incisor. It was because somebody had taken out other teeth to such an extent that they could not be brought into proper occlusion. I am very much astonished that Dr. Baker succeeded as he did in the case where the first molars were gone.

Regarding the question of the teeth not being adapted in size to the mouth they occupy, my answer is this: that the teeth are not more perfect than the rest of the body; some people are born with a club foot and some with dislocated hips. Dr. Ottolingui says that he hears a great deal about big teeth of big father and little jaw of little mother, but he has never heard of a big jaw from a big father and little teeth from a little mother. The fact is that the teeth of each individual are adapted to that individual and his size. I know of only one man who has attempted accurately to measure the teeth. This man is Dr. Hawley. I think his object was to find a method of forming the normal arch from the sizes of two erupted teeth. He has shown how we may arrange the whole arch from *three* teeth, a central, lateral and cuspid.

A vote of thanks was extended to Dr. Baker for his helpful demonstration.

Adjourned.

FRED L. BOGUE, M.D., D.D.S.,
*Editor The New York Institute of Stomatology.*

